# Supplementary material for: Predicting visual function by interpreting a neuronal wiring diagram
Source: Nature. 2024 Oct 2;634(8032):113–23. doi: 10.1038/s41586-024-07953-5 (PMC11446822; doi:10.1038/s41586-024-07953-5)
Supplement: Supplementary file 6 — CRF and ERF predictions for individual TmY4 and TmY9 cells. Analogous to Supplementary Data 3, but for TmY target types. Shown are the top four monosynaptic pathways, the strongest pathway passing through each of the top ten intermediary types (ranking from Extended Data Fig. 7), and the trisynaptic pathway Tm1–TmY–Dm3–TmY (see the section entitled Prediction of spatial normalization). [file 41586_2024_7953_MOESM6_ESM.zip › DataS4/TmY9q/720575940617118395.pdf]

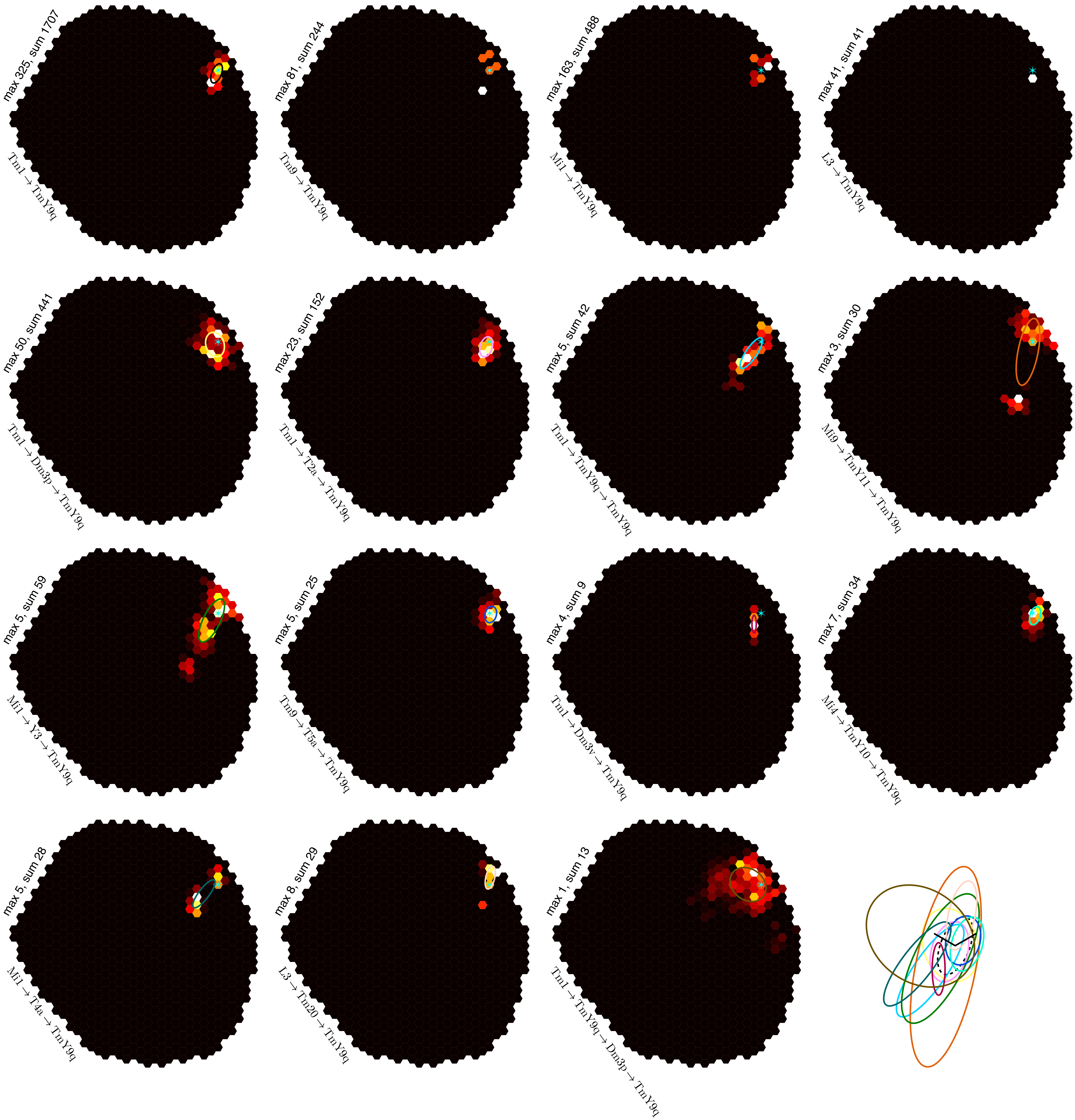
max 325, sum 1707
Tm1 → TmY9q
max 81, sum 244
Tm9 → TmY9q
max 163, sum 488
Mi1 → TmY9q
max 41, sum 41
L3 → TmY9q
max 50, sum 441
Tm1 → Dm3p → TmY9q
max 23, sum 152
Tm1 → T2a → TmY9q
max 5, sum 42
Tm1 → TmY9q → TmY9q
max 3, sum 30
Mi9 → TmY11 → TmY9q
max 5, sum 59
Mi1 → Y3 → TmY9q
max 5, sum 25
Tm9 → T5a → TmY9q
max 4, sum 9
Tm1 → Dm3v → TmY9q
max 7, sum 34
Mi4 → TmY10 → TmY9q
max 5, sum 28
Mi1 → T4a → TmY9q
max 8, sum 29
L3 → Tm20 → TmY9q
max 1, sum 13
Tm1 → TmY9q → Dm3p → TmY9q
